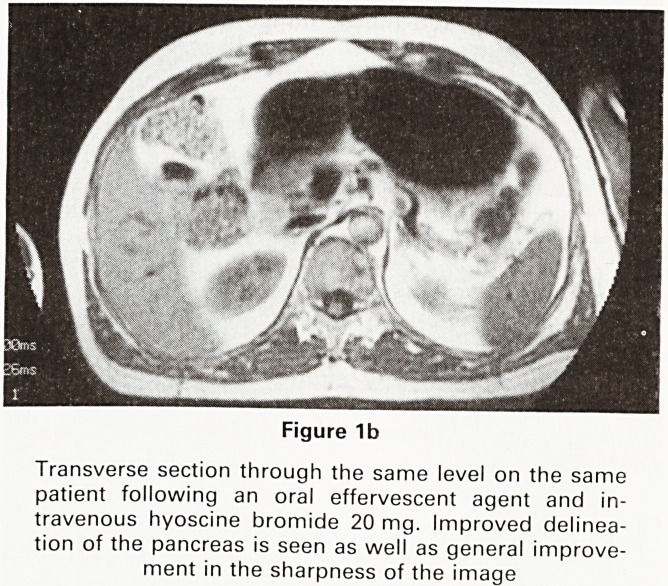# Magnetic Resonance Imaging of the Pancreas

**Published:** 1988-05

**Authors:** J. Kabala, J. Virjee, R. Mountford, J. Waring, R. Yeats, P. Goddard


### Magnetic Resonance Imaging of the Pancreas

**J. Kabala MRCP, J. Virjee FRCR, R. Mountford MD MRCP FRCR, J. Waring, R. Yeats, P. Goddard MD FRCR**

Magnetic Resonance Imaging (MRI) can regularly provide good images of the pancreas (1,2). Medium and large intra-abdominal blood vessels are well seen with MRI due to the high contrast between solid structures and the low or absent signal intensity of flowing blood (1–5). The pancreas is delineated by the splenic and portal veins posteriorly and the retrogastric fat anteriorly (6,7). The quality of the images of the pancreas and nearby structures (the kidneys and adrenals, splenic and portal veins, the superior mesenteric artery and the coeliac axis) is influenced by a number of factors.

1. In common with computed tomography, demonstration of the retroperitoneal organs is generally better in patients with ample intra-abdominal fat (1). The pancreas may be incompletely defined in the occasional patient with virtually no intra-abdominal fat (2).

2. The distribution and/or absence of gas in the upper gastro-intestinal tract can influence visualisation of the pancreas with MRI (1,6). Pancreas and gasless bowel have similar $T_1$ and $T_2$ values (1,6,7). Oral paramagnetic agents such as ferric ammonium nitrate have been used to alter relaxation times of proximal foregut (6,7) and produce different signal intensities from bowel loops. The use of an oral effervescent agent immediately before the scan (producing gas in the stomach and duodenal loop) is cheaper and likely to be as effective.

3. MRI images, even more than CT, are degraded by patient movement. In the abdomen respiratory movement, blood flow and peristalsis can produce problems (1,2,4), in particular blurring and/or ghost images. Respiratory gating is available but prolongs scan times. Alternative software exists to try and overcome this problem. For example, respiratory phase encoding (ROPE) relies on a mechanical device to monitor the patient's respiration. ROPE does not reject any data and consequently does not increase scan times (6).

Movement artefact suppression (MAST) sequences are useful to eliminate artefacts due to predictable and repeated motion as in blood flow. Glucagon or an anticholinergic agent given intravenously to inhibit peristalsis can be seen to improve images of the pancreas (Figures 1a and b)

### CONCLUSION

Currently in Bristol the pancreas is imaged in the coronal and transverse plane with a $T_1$ weighted spin echo sequence (TR=500ms, TE=26 ms). An additional MAST sequence with $T_2$ weighting (TR=1800 ms, TE=100ms) is also performed in the coronal plane. The $TR_{500}$ $TE_{26}$ sequence takes 7.6 minutes each for the coronal and transverse scans, the $TR_{1800}$ $TE_{100}$ takes 11.5 minutes, giving a total scan time of 26.7 minutes. These sequences provide good visualisation of other retroperitoneal structures[16] as well as the pancreas and its related vasculature. Data is collected using a multislice technique with the patient performing quiet regular respiration. Peristalsis is inhibited by giving hyoscine bromide 20 mg intravenously immediately prior to the scan (unless contra-indicated by the presence of heart disease or glaucoma, in which case glucagon 0.3 mg is used). An oral effervescent agent is given at the same time. This is the first time this combination of sequences and techniques has been used regularly for pancreatic MRI.

**Figure 1a**

Transverse section through the abdomen at the level of the head of pancreas. $TR_{500ms}$ $TE_{26\ ms}$

**Figure 1b**

Transverse section through the same level on the same patient following an oral effervescent agent and intravenous hyoscine bromide 20 mg. Improved delineation of the pancreas is seen as well as general improvement in the sharpness of the image

### REFERENCES

1. STARK, D. J. MOSS, A. A. GOLDBERG, H. I. et al (1984) Magnetic resonance and CT of the normal and diseased pancreas: a comparative study. *Radiology* **150**, 153–162.
2. TSCHOLAKOFF, D. HRICK, H. THOENI, R. et al (1988) MR imaging in the diagnosis of pancreatic disease. *AJR* **148**, 703–9.
3. ALFIDI, R. J. HAAGA J. R. ELYOUSEF, S. J. et al (1982) Preliminary experimental results in humans and animals with a superconducting whole-body nuclear magnetic resonance scanner. *Radiology* **143**, 175–181.
4. BUONCORE, E. BONKOWSKI, G. P. PAVLICEK, W. NGO, F. (1983) NMR imaging of the retroperitoneum: Technical considerations. *AJR* **14**, 1171–1178.
5. HIGGINS, C. B. GOLDBERG, H. HRICAK, H. et al (1983a) Nuclear magnetic resonance imaging of vasculature of abdominal viscera: Normal and pathological features. *AJR* **140**, 1217–1225.
6. GRAIF, M. LEUNG, A.W.-L. STEINER, R. E. YOUNG, I. R. (1986) Magnetic resonance imaging of the retroperitoneum. *Clinical Radiology* **37**, 441–449.
7. BYDDER, G. M. STEINER, R. E. WORTHINGTON, B. S. in Diagnostic radiology, R. G. Grainger, and D. J. Allison (1986) Churchill Livingstone (London).

## Figures and Tables

**Figure 1a f1:**
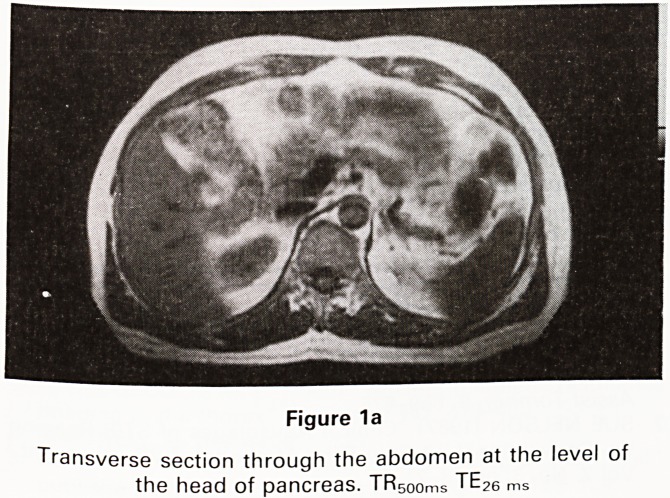


**Figure 1b f2:**